# From capability uplift to capability governance: an AI–biosecurity stack

**DOI:** 10.3389/fmicb.2026.1899413

**Published:** 2026-08-14

**Authors:** Lyly Luhachack, Nancy Connell, Kavita Berger

**Affiliations:** 1 National Academies of Sciences, Engineering, and Medicine, Washington, DC, United States; 2 Department of Medicine, Rutgers New Jersey Medical School, Newark, NJ, United States

**Keywords:** agentic AI, artificial intelligence, autonomous laboratories, biological foundation models, biosecurity, biotechnology governance, synthetic biology

## Abstract

Artificial intelligence is becoming a general-purpose enabling technology for the life sciences. AI-enabled tools can strengthen biosecurity and biodefense by improving early warning, accelerating vaccine and therapeutic discovery, supporting laboratory safety, and enabling more adaptive preparedness systems. However, these same tools may be a potential amplifier of misuse. The 2025 National Academies’ report *The Age of AI in the Life Sciences: Benefits and Biosecurity Considerations* provides an important foundation for this analysis of the “capability uplift” enabled by AI across the design–build–test–learn (DBTL) cycle. “Capability uplift,” or ΔAI, is a term used in the report to assess how AI-enabled biological tools can uniquely, and in some cases, specifically enable increases in or changes to biosecurity risks. The report proposes an “if–then” approach to monitor emerging capabilities through observable indicators such as new datasets as the leading indicator of capability, model performance, and the erosion of build/test barriers. Since the report’s publication, agentic AI systems, virtual scientific teams, genome-scale foundation models, and self-driving laboratories have advanced from largely prospective concerns to early demonstrations. Multi-agent systems have been reported for biomedical hypothesis generation, design, and semi-autonomous discovery workflows, while self-driving laboratories now are considered as practical platforms for biotechnology. This perspective article extends these insights by employing a conceptual framework analysis and involves: (1) categorizing key AI capabilities across the DBTL cycle into a layered capability stack, and (2) illustrate how the if-then approach can be used to inform a dashboard based on observable indicators.

## 1 Introduction

The convergence of AI and the life sciences has generated two competing narratives. One emphasizes discovery and acceleration: AI can help interpret biological data (e.g., genomic, transcriptomic, proteomic, epidemiological, clinical, environmental), design proteins, prioritize antimicrobial candidates, improve biosurveillance, and speed medical countermeasure development. The other emphasizes misuse: AI could lower barriers for designing or modifying harmful biological agents. The National Academies of Sciences, Engineering, and Medicine report, *The Age of AI in the Life Sciences: Benefits and Biosecurity Considerations*, frames the tensions between these two narratives by examining what AI changes in the biological workflow and where that change matters for biosecurity (National Academies of Sciences, Engineering, and Medicine, 2025). It also highlights the concept of AI-enabled capability uplift, or ΔAI, and concludes that the strongest current uplift is in ideation and design while experimental validation remains a major bottleneck (National Academies of Sciences, Engineering, and Medicine, 2025). This analysis can be reframed as a capability stack for AI-enabled biology to highlight that no single model, database, or laboratory platform determines risk independently. Risk and benefit emerge from connections among data, models, agents, laboratory automation, synthesis access, experimental validation, and governance.

The frontier of AI and biology has shifted from isolated biological design tools toward integrated systems, making the timing of this framing especially timely. Agentic AI systems can now distribute tasks among specialized agents, generate and critique hypotheses, plan analyses, and coordinate scientific workflows (Xin et al., 2025; Dip et al., 2026; Huynh et al., 2026; Li et al., 2026; Marx, 2026). For example, Ghareeb et al. (2026), developed Robin, a multi-agent system integrating literature-search and data-analysis agents to generate hypotheses, propose experiments, interpret results, and update hypotheses in an experimental-biology workflow.

## 2 The AI-biology capability stack

The 2025 National Academies’ report assessed the capability uplift of AI-enabled biological tools across the design-build-test-learn (DBTL) cycle (2025). A stack-based approach can help conceptualize this capability assessment as an integrated system. In computing, a collection of components, or layered stacks, that build on one another to provide a complete system can clarify dependencies among hardware, infrastructure, models, data, and applications. Analogously, a capability stack for AI systems in biology that consists of layers including foundational data, models, and other components can help to identify the building blocks from which future biological AI capabilities may emerge (Table 1). Viewing AI for biology through a capability-stack lens shifts attention from the properties of individual layers to the capabilities that emerge when those components are combined. For biosecurity and biodefense, the relevant question is whether the coupling of layers produces new speed, scale, access, or autonomy.

**Table 1:** Biosecurity-focused AI capability stack.

Layer	Recent advances	Application	Concern	Possible governance strategies
Data and provenance	Larger curated biological datasets, including genome-scale resources; National Academies identifies data availability as a leading indicator of AI capability.	Better microbial annotation, variant interpretation, surveillance, and countermeasure design.	Noisy, biased, poisoned, or poorly contextualized data may distort models or enable unanticipated capabilities.	Data provenance, repository curation, tiered access.
Biological foundation models	Evo 2 was reported as a genome model trained on about 9 trillion DNA base pairs with a 1-million-token context window across domains of life (Brixi et al., 2026)	Prediction across microbial, phage, and host-associated sequence contexts.	More general models may improve long-context reasoning over genomes.	Capability evaluations, model documentation, access logs
Agentic AI	Multi-agent systems now perform literature review, hypothesis generation, debate, tool selection, and data analysis in biomedical contexts (Xin et al., 2025; Li et al., 2026; Marx, 2026)	Accelerates interdisciplinary workflows	Agents may reduce tacit-knowledge barriers by translating broad goals into executable workflows.	Agent audit logs, tool permissioning, human approval gates, red-team testing.
Build/test automation	Self-driving labs are being developed for biotechnology but remain complex and use-case dependent (Canty et al., 2025; Collins et al., 2025; Tobias and Wahab, 2025)	Faster closed-loop optimization, reproducibility, and dataset generation.	Reduced build/test friction may erode a major biosecurity bottleneck.	Protocol whitelists, inventory gates, anomaly detection, biosafety constraints,
Public health and response layer	AI tools as part of institutionalized preparedness infrastructure (Centers for Disease Control and Prevention, 2026; World Economic Forum, 2026, OpenAI, 2026)	Earlier detection, faster risk assessment, and accelerated medical countermeasures.	Overly restrictive controls could slow preparedness.	Emergency partnerships, pre-cleared models, secure compute, public–private response triggers.

At the data layer, prioritizing AI-ready, context-rich datasets is critical. The goal is not simply more sequences, but better-linked data (e.g., organism, environment, phenotype, assay conditions, uncertainty) with provenance and permissible use. Further, national repositories and data stewardship are foundational for AI-enabled biology (National Academies of Sciences, Engineering, and Medicine, 2025).

At the model layer, biology needs benchmarks that reflect real biological complexity. Many AI evaluations overemphasize static sequence or structure tasks. Useful benchmarks test generalization of models. Evaluating models for beneficial capabilities, such as outbreak triage or AMR (antimicrobial resistance) prediction, and dual-use-relevant capabilities, such as whether they infer traits that should trigger added biosecurity review, are a critical part of responsible innovation.

At the agent layer, AI systems treated as workflow participants, rather than as chatbots, add greater scientific value. Agentic systems can read papers, write code, select tools, summarize data, and propose experiments. The critical question is not only what the model knows, but what the agent can do. An agent with no tool access may require ordinary responsible-use guidance, whereas an agent with access to data repositories, cloud labs, synthesis ordering, or robotic instruments may require formal controls.

At the laboratory layer, autonomous labs can help to accelerate experimental work. Yet increased experimental capability does not imply uniform risk across applications. For example, low-risk closed-loop optimization of enzyme expression is not equivalent to open-ended work involving pathogenic organisms or poorly characterized environmental isolates. The build-test stages remain a bottleneck and are a critical consideration for governance and oversight (National Academies of Sciences, Engineering, and Medicine, 2025).

The capability stack then highlights how capabilities build upon one another. It also highlights that coupling of a model with agentic interface, cloud lab, and permissive data access may be the most consequential change versus any single layer. This same coupling can transform beneficial biosecurity applications. A public health agency and researchers with secure access to curated microbial data, validated models, and pre-approved laboratory workflows could move from detection to candidate countermeasure design faster than current systems allow. This structure is intended to be practical rather than exhaustive, allowing policymakers, funders, biosecurity practitioners, model developers, journal editors, institutional biosafety committees, and public-health agencies to ask questions such as, what layer does a capability occupy, what biological outputs can it generate, how close is it to physical execution, what data does it require, how autonomous is it, and what safeguards are appropriate.

### 2.1 Capability stack: recent advances

The National Academies’ report highlighted agentic AI, scientific large language models, automated laboratories, and synthetic datasets as a few areas to watch for emerging developments (National Academies of Sciences, Engineering, and Medicine, 2025). Since then, a few notable developments have emerged in those areas.

Agentic AI has become a practical interface layer. Future AI agents have the potential to reduce expertise barriers (National Academies of Sciences, Engineering, and Medicine, 2025). The frontier of foundation models has moved toward multi-agent systems that assign roles, debate hypotheses, select tools, and interact with experimental or computational pipelines. For example, the Virtual Lab demonstrates a new organizational form of an AI-mediated team science with a human providing high-level feedback (Swanson et al., 2025). This development suggests that future capability uplift may arise from orchestration rather than from model output alone.

Biological foundation models are becoming more genome-scale and multimodal. In late 2025, Brixi et al., released Evo 2, which extends biological language modeling to long genomic contexts across domains of life, with model parameters, training code, inference code, and the OpenGenome2 dataset (Brixi et al., 2026). Although Evo 2 illustrates the rapid pace with which biological models are advancing, this capability alone does not mean such models can now make the leap to designing dangerous novel pathogens. However, genome-scale models may change the monitoring problem, highlighting the importance of capabilities uplift assessments to track long-context genomic reasoning, mobile-element interpretation, host–microbe prediction, and microbial fitness forecasting in addition to protein design benchmarks.

Autonomous laboratories are becoming a near-term governance issue. Fully self-driving laboratories remained a future capability with significant challenges, including application specificity, high resources, human input, and capital investment (National Academies of Sciences, Engineering, and Medicine, 2025). Interest in developing self-driving biotechnology labs has led to both public and private sector investments (National Science Foundation, 2025; Department of Energy, 2026). This suggests a shift from asking whether self-driving labs can be built to asking which tasks they can automate, under which biosafety constraints, and with what auditability.

## 3 Applying the if–then strategy to AI-biosecurity

The National Academies’ report recommended an if–then strategy that avoids speculative prediction by linking observable developments to capability assessments and evaluation for potential biosecurity risks. Table 2 provides illustrative examples of how this strategy could be applied to AI-enabled biosecurity that could prompt evaluation questions and proportionate governance responses in developing an operational dashboard.

**Table 2:** Examples of the If-then strategy

If this observable appears…	Then evaluate…	Then implement…
Agentic systems can autonomously turn high-level biological goals into multi-step computational and wet-lab workflows.	Does the system merely suggest ideas, or can it invoke tools, order materials, control instruments, or send jobs to cloud labs?	Tool permissioning, human approval before external actions, tamper-evident logs, institutional review of agent workflows.
Models show strong prediction of microbial phenotype from genotype across diverse organisms or conditions.	Are predictions limited to benign traits, or do they generalize to virulence, transmissibility, host range, immune escape, or resistance?	Sandboxed evaluation, controlled benchmark access, dataset review, benefit–risk assessment before broad release.
Genome-scale models incorporate long-context microbial, phage, plasmid, and host-associated data.	Does performance improve on mobile genetic elements, host–microbe interactions, or microbial fitness landscapes?	Tiered access for sensitive weights or fine-tunes, secure red-team evaluation.
Biomolecular design tools generate functional molecules with low sequence homology to known sequences.	Can current nucleic acid screening detect these designs? Are structural or functional similarities missed?	Structure/function-aware screening, updated sequences-of-concern logic, know-your-order metadata, provider engagement.
Closed-loop labs reduce build/test cycles for microbial or biochemical design.	Which assays are automated? Are organisms, toxins, virulence factors, or delivery systems involved?	Whitelisted protocols, biosafety interlocks, inventory controls, anomaly detection, independent audits, digital twins.
AI-generated synthetic data become part of model training for microbiology.	Does synthetic data improve performance or cause model collapse, bias, or hidden failure modes?	Synthetic-data labeling, provenance requirements, stress testing, retraining audits.
AI systems materially shorten time from pathogen detection to countermeasure candidate.	Is the improvement reproducible, validated, and safely deployable during an emergency?	Pre-cleared emergency partnerships, secure compute access, regulatory handoff protocols, international coordination.

The if-then strategy, and illustrative examples in Table 2, are not intended to be a binary alarm system. Each row may consider dimensions such as severity, confidence, reversibility, benefit, and responsible actors before being used in an operational setting. For example, an agent that drafts a literature review requires different controls than an agent with API access to a cloud laboratory. Similarly, a protein model that designs benign binders requires different controls than a model demonstrating robust prediction of traits linked to pathogenicity. These examples can serve as a starting point for what factors to consider in developing future operational dashboards.

## 4 Governance principles

The capability stack and if-then strategy indicate several governance principles to consider. First, governance based on consideration of capabilities is more effective than labels. “AI,” “foundation model,” and “agent” are broad terms. Governance of the interfaces of the capability stack may provide a strategic approach versus a single layer alone. A biological model behind a chat interface, an API (application programming interface), a lab robot, and a synthesis-ordering system carries different risks and therefore, requires different approaches to governance and oversight. AI-enabled tools can strengthen preparedness and response by improving biosurveillance, diagnostics, vaccine design, antibody development, and antimicrobial discovery. Overly broad restrictions may slow precisely the capabilities needed for outbreak response. Fourth, open data and open models are powerful drivers of science, requiring consideration of the consequences of graduated access. Some combinations of model capability, dataset sensitivity, and tool access may be considered for determining options such as controlled release, registered access, secure environments, or sandboxed evaluation. Finally, to be effective, AI-biosecurity governance cannot be a one-time review but rather a continuous assessment that would allow for assessment of risk and benefit as new capabilities are developed. Models improve, datasets expand, agents gain tools, and laboratories become more automated.

Implementing capability-based AI-biosecurity governance would involve practical challenges that are not fully addressed by the capability stack alone. These implementation challenges include the cost of continuous evaluation, time required to develop and update benchmarks, technical capacity to audit models and agentic systems, and the coordination required across the relevant research and development ecosystem. Acknowledgement of these challenges and obstacles is important as governance recommendations may be difficult to operationalize or place uneven burden on different actors. Future work that examines how capability evaluations, access controls, audit mechanisms, and response triggers could be resourced and coordinated may help address feasibility issues.

## 5 Discussion

The National Academies’ report offers a method for dynamic evaluation of AI capability uplift in relation to biological complexity, data availability, and the DBTL bottleneck. This perspective extends this method into a capability stack for biosecurity and biodefense combined with an if–then framework that could be further developed and operationalized. The capability stack and if-then approach proposed here have several limitations. First, they are intended as conceptual and governance planning tools rather than complete risk-assessment methods or implementation plans. The stack helps organize where AI-enabled biological capabilities may emerge but does not by itself determine severity, likelihood, or tolerance of a particular risk. Similarly, the if-then examples illustrate how observable developments could prompt further evaluation but do not establish validated thresholds or prescribe specific decisions. In addition, this approach depends on the availability and interpretation of observable indicators. Finally, coordination and capacity across multiple actors is required and capability evaluations and governance triggers may be difficult to implement without resources.

Future research may include: (1) empirical testing of an if-then dashboard in real-world biosecurity settings; (2) developing quantitative metrics for each layer of the capability stacks; (3) assessing the cost-effectiveness of potential governance measures; (4) assessing institutional capacity for implementing oversight recommendations; and (5) considering the ability of governance regimes of other emerging technology to handle similar uncertainties.

Since the publication of the report, the frontier is increasingly agentic, integrated, and automated. Multi-agent systems can coordinate scientific work; genome models can reason over longer biological contexts; design tools can generate experimentally validated biomolecules; and self-driving laboratories are becoming practical in selected biotechnology workflows. These developments make governance more urgent at the interfaces of the stack.

Reframing the current AI biosecurity debate as capability governance accounts for the complexity of the system. A capability stack plus if-then approach offers a practical path of identifying where capability is emerging, specifying what would trigger concern, and predefining proportionate actions that protect both security and scientific progress.

## Data Availability

Publicly available datasets were analyzed in this study. This data can be found at: https://doi.org/10.17226/28868.
